# Dynamic nesting of *Anaplasma marginale* in the microbial communities of *Rhipicephalus microplus*

**DOI:** 10.1002/ece3.11228

**Published:** 2024-04-01

**Authors:** Elianne Piloto‐Sardiñas, Lianet Abuin‐Denis, Apolline Maitre, Angélique Foucault‐Simonin, Belkis Corona‐González, Cristian Díaz‐Corona, Lisset Roblejo‐Arias, Lourdes Mateos‐Hernández, Roxana Marrero‐Perera, Dasiel Obregon, Karolína Svobodová, Alejandra Wu‐Chuang, Alejandro Cabezas‐Cruz

**Affiliations:** ^1^ ANSES, INRAE, Ecole Nationale Vétérinaire d'Alfort, UMR BIPAR, Laboratoire de Santé Animale Maisons‐Alfort France; ^2^ Direction of Animal Health, National Center for Animal and Plant Health Carretera de Tapaste y Autopista Nacional San José de las Lajas Cuba; ^3^ Animal Biotechnology Department Center for Genetic Engineering and Biotechnology Havana Cuba; ^4^ INRAE, UR 0045 Laboratoire de Recherches Sur Le Développement de L'Elevage (SELMET‐LRDE) Corte France; ^5^ EA 7310, Laboratoire de Virologie, Université de Corse Corte France; ^6^ School of Environmental Sciences University of Guelph Guelph Ontario Canada; ^7^ Faculty of Science University of South Bohemia Ceske Budejovice Czech Republic

**Keywords:** *Anaplasma marginale*, microbiome dynamics, nesting, networks, *Rhipicephalus microplus*, ticks

## Abstract

Interactions within the tick microbiome involving symbionts, commensals, and tick‐borne pathogens (TBPs) play a pivotal role in disease ecology. This study explored temporal changes in the microbiome of *Rhipicephalus microplus*, an important cattle tick vector, focusing on its interaction with *Anaplasma marginale*. To overcome limitations inherent in sampling methods relying on questing ticks, which may not consistently reflect pathogen presence due to variations in exposure to infected hosts in nature, our study focused on ticks fed on chronically infected cattle. This approach ensures continuous pathogen exposure, providing a more comprehensive understanding of the nesting patterns of *A. marginale* in the *R. microplus* microbiome. Using next‐generation sequencing, microbiome dynamics were characterized over 2 years, revealing significant shifts in diversity, composition, and abundance. *Anaplasma marginale* exhibited varying associations, with its increased abundance correlating with reduced microbial diversity. Co‐occurrence networks demonstrated *Anaplasma*'s evolving role, transitioning from diverse connections to keystone taxa status. An integrative approach involving *in silico* node removal unveils the impact of *Anaplasma* on network stability, highlighting its role in conferring robustness to the microbial community. This study provides insights into the intricate interplay between the tick microbiome and *A. marginale*, shedding light on potential avenues for controlling bovine anaplasmosis through microbiome manipulation.

## Graphical abstract

This study investigates the dynamic interactions within the microbiome of the cattle tick *Rhipicephalus microplus*, emphasizing its relationship with the tick‐borne pathogen *Anaplasma marginale*. By analyzing ticks feeding on chronically infected cattle over a two‐year period using next‐generation sequencing, the research reveals significant fluctuations in microbiome diversity, composition, and abundance. The study elucidates the evolving role of *A. marginale* within the microbiome, highlighting its impact on microbial community stability and suggesting potential strategies for controlling bovine anaplasmosis through microbiome manipulation.

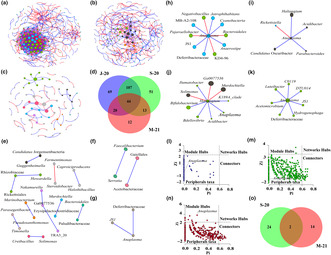

## 1 INTRODUCTION

Interactions between symbionts, commensals, and tick‐borne pathogens (TBPs) within the tick microbiome can potentially shape disease ecology. Symbiont–microbiome interactions in questing *Ixodes ricinus* can be dynamic, with variations observed in the prevalence and distribution of tick symbionts across different forest sites (Krawczyk et al., 2022). The strongest determinants of microbiome clustering were found to be the abundance and prevalence of specific symbionts, such as *Rickettsia* and *Rickettsiella*. The proportions of these symbionts varied between geographically close forest sites, suggesting a potential spatial scale influencing their distribution. The observed variations in the prevalence of tick symbionts were not consistent with horizontally transmitted pathogens such as *Borrelia afzelii*, *Borrelia garinii*, *Anaplasma phagocytophilum*, and *Neorickettsia mikurensis*, which showed more random patterns across geographically close forest sites (Krawczyk et al., 2022). A finding supported by previous studies in different tick species including *I. ricinus* (Lejal et al., 2019; Zając et al., 2023), *Dermacentor reticulatus* (Zając et al., 2023), and *Rhipicephalus microplus* (Piloto‐Sardiñas, Foucault‐Simonin, et al., 2023). This distinction suggests that the factors influencing symbiont prevalence may differ from those affecting horizontally transmitted pathogens, which are mainly determined by local vertebrate communities (Takumi et al., 2019).

Microbe–microbe associations within the tick microbiome, particularly those involving TBPs and other nonpathogenic bacteria, exhibit dynamic patterns over time (Lejal et al., 2021). The temporal dynamics of the *I. ricinus* microbiome and its impact on microbiome–pathogen interactions were evaluated in questing ticks collected during three consecutive years in a peri‐urban forest in France (Lejal et al., 2021). Results revealed temporal variations in the microbiome, with distinct clusters of tick samples collected during different months. *Wolbachia*, *Arsenophonus*, *Spiroplasma*, and *Pseudomonas* were identified as drivers of certain clusters, indicating their role in shaping temporal variations (Lejal et al., 2021). Notably, comparisons of tick samples positive for specific TBPs (*Rickettsia*, *Borrelia*, and *Anaplasma*) with TBP‐negative samples demonstrated significantly higher abundance of relevant operational taxonomic units (OTUs) in TBP‐positive samples. Network analyses revealed varying correlation patterns between TBP‐positive and TBP‐negative samples, with *Borrelia*‐positive samples showing both positive and negative correlations with specific OTUs, including those associated with environmental and pathogenic/symbiotic genera (Lejal et al., 2021).

Temporal changes in the vector microbiome may affect the ability of pathogens to persist in vectors, transmit to hosts, and cause disease, as the transmission of vector‐borne pathogens often involves complex interactions between pathogens and the microbiome (Abraham et al., 2017; Maitre et al., 2022, 2023; Narasimhan et al., 2014, 2017). While the studies by Krawczyk et al. (2022), and Lejal et al. (2021) provided valuable insights into the dynamic nature of symbiont–microbiome interactions and the temporal dynamics of TBPs within questing ticks, it is essential to acknowledge a crucial limitation inherent to this sampling approach. The absence of a pathogen in a questing tick may be linked to the fact that the host on which the tick fed did not harbor the pathogen in the first place (Takumi et al., 2019). This inherent limitation poses a challenge in disentangling whether the absence of a TBP in a tick is due to the absence of the pathogen in the previous host or influenced by the tick microbiome.

This sampling strategy also prevents assessing nesting dynamics of TBPs in the tick microbiome, as dosage and frequency of pathogen exposure are difficult to control in natural settings. To address the challenge associated with the limitations of questing tick sampling and better elucidate TBP–microbiome interactions in nature, we propose exploring systems involving ticks feeding on chronically infected hosts. In such scenarios, ticks would encounter a continuous presence of the pathogen across time. This approach, exemplified by systems such as *R. microplus* feeding on cattle chronically infected with *Anaplasma marginale*, provides an unique opportunity to assess the impact of the microbiome on TBP dynamics under more controlled conditions.

*Rhipicephalus microplus*, commonly known as the cattle tick or tropical cattle tick, is a significant ectoparasite that infests cattle and other livestock. This tick species holds great importance due to its capacity to transmit various pathogens, with *A. marginale* being one of the most notable (De La Fourniere et al., 2023; Pereira et al., 2022). *Anaplasma marginale* is a bacterium that causes bovine anaplasmosis, a disease characterized by anemia, fever, and other clinical symptoms in cattle (Salinas‐Estrella et al., 2022). This disease can lead to significant economic losses due to decreased productivity, increased veterinary costs, and even livestock mortality (Rodríguez et al., 2009; Salinas‐Estrella et al., 2022). *Rhipicephalus microplus* plays a crucial role in the transmission of *A. marginale*, as it acts as a vector by feeding on infected cattle and subsequently transmitting the bacteria to susceptible animals during subsequent feedings (Zivkovic et al., 2010). In a recent study, it was observed that while *A. marginale* consistently infected all cattle across different sampling periods, its presence was not uniformly detected in all *R. microplus* infesting the cattle (Piloto‐Sardiñas, Foucault‐Simonin, et al., 2023). This indicates nonlinearity between tick infestation rate and pathogen prevalence in ticks (Ostfeld & Keesing, 2023), likely under strong influence of the tick microbiome (Tonk‐Rügen et al., 2023).

The present study aimed to investigate whether temporal fluctuations in the microbial communities of the cattle tick *R. microplus* could potentially disturb or alter the interactions between *A. marginale* and the tick microbial communities and as a consequence shape the impact of the pathogen on the microbial community assembly. To achieve this, *R. microplus* samples confirmed for infection with *A. marginale* were used (Piloto‐Sardiñas, Foucault‐Simonin, et al., 2023), and next‐generation sequencing (NGS) was performed for microbiome characterization. Employing an experimental network approach, an *in silico* node removal technique was applied, a strategy previously employed to investigate the impact of *Rickettsia* pathogens on the microbiome assembly in *Hyalomma marginatum* and *Rhipicephalus bursa* ticks (Maitre et al., 2023). By simulating the absence of specific TBPs such as *Anaplasma* sp. *in silico*, the objective was to evaluate the impact on clustering patterns, microbial composition and abundance of diverse taxa, community assembly, and network robustness over time. With this integrative approach, we aimed to uncover connections between *A. marginale* presence, temporal dynamics of the microbiome, and network structure, potentially identifying key microbial taxa to be used in anti‐microbiota vaccines for the control of bovine anaplasmosis.

## 2 MATERIALS AND METHODS

### 2.1 Study design and tick samples

Tick samples collected from eight bovines on a farm in Mayabeque province, Cuba (Piloto‐Sardiñas, Foucault‐Simonin, et al., 2023) at three time points: July 2020 (J‐20), September 2020 (S‐20), and March 2021 (M‐21) were included in this study. Engorged adult female ticks were manually collected from the same animals at different time points and morphologically identified as *R. microplus* using standardized taxonomic keys (Estrada‐Peña et al., 2004; Piloto‐Sardiñas, Foucault‐Simonin, et al., 2023). Tick‐borne pathogens (TBPs) were detected in individual tick samples through high‐throughput real‐time microfluidic PCR method (Piloto‐Sardiñas, Foucault‐Simonin, et al., 2023). This PCR method allows the detection of 27 bacterial species (belonging to the bacterial genera *Borrelia*, *Anaplasma*, *Ehrlichia*, *Rickettsia*, and *Mycoplasma*), 7 parasite species (such as *Babesia* and *Hepatozoon*), 5 bacterial genera, and 3 parasites taxa (Apicomplexa, *Theileria* and *Hepatozoon*) (Grech‐Angelini et al., 2020; Michelet et al., 2014). The tested pathogens, target genes, and primer sequences used for amplification are shown in Table S1 (Gondard et al., 2020). Tick samples showing a low level of engorgement and single *A. marginale* infection (Piloto‐Sardiñas, Foucault‐Simonin, et al., 2023) were selected for microbiome sequencing.

Before DNA extraction, the collected ticks underwent a washing process, which involved two rounds of washing in miliQ sterile water and one round in 70% ethanol. It is worth noting that ethanol, rather than bleach, was used for washing to intentionally include both internal and external tick microbiome in our analysis, as we consider tick surface microbes to be part of the tick's microbiome. Following the washing process, the ticks were preserved in 70% ethanol and stored at −80°C until further processing. For the extraction of total DNA, the homogenization of whole ticks was performed on a MagNA Lyser instrument (Roche Molecular Diagnostics, Rotkreuz, Switzerland) at a speed of 5000 rpm for 5 cycles of 60 s each. Total DNA extraction was performed using the Wizard Genomic DNA Purification kit (Promega, Madison, WI, USA) according to the manufacturer's instructions. The DNA samples were eluted in 60 μL of DNA Rehydration Solution. The used of Colibri Microvolume Spectrophotometer (Titertek‐Berthold, Pforzheim, Germany) allowed determining the quantitative and qualitative assessment of DNA extraction. Reagent extraction controls were set in DNA extraction process, using the same conditions as for the samples but using water as template. DNA amplification was then performed on the extraction control in the same conditions as for any other sample.

### 2.2 16S rRNA amplicon sequencing and processing of raw sequences

A single lane of the Illumina MiSeq system was used to generate 251‐base paired‐end reads from variable region V4 of the 16S rRNA gene using barcoded universal primers (515F/806R) in ticks. The paired 16S rRNA raw sequences obtained from the J‐20 (*n* = 7), S‐20 (*n* = 7), and M‐21 (*n* = 8) samples were deposited in the SRA repository (Bioproject No. PRJNA1028823). Analysis of 16S rRNA sequences was performed using the Quantitative Insights into Microbial Ecology 2 (QIIME 2) pipeline (v. 2021.4) (Bolyen et al., 2019). The raw sequences (demultiplexed in fatsq files) were denoized, quality trimmed, and merged using the DADA2 software (Callahan et al., 2016) implemented in QIIME2 (Bolyen et al., 2019). The obtained amplicon sequence variants (ASVs) were aligned with q2‐alignment of MAFFT (Katoh et al., 2002) and used to generate a phylogeny with q2‐phylogeny of FastTree 2 (Price et al., 2010). Taxonomy was assigned to ASVs using a classify‐sklearn naïve Bayes taxonomic classifier based on SILVA database (release 138) (Bokulich et al., 2018). Only the target sequence fragments were used for the classifier (i.e., the classifier was trained with primers 515F/806R) (Ren & Wu, 2016; Werner et al., 2012).

### 2.3 Identification and removal of contaminants

The possible contaminants in the samples were statistically identified with the “Decontam” (Davis et al., 2018) package using the “prevalence” method. The method used compares the prevalence of each sequence feature in true samples to the prevalence in negative controls from the DNA extraction process to identify contaminants. Then, contaminants were removed from the dataset before downstream microbiome analysis (Davis et al., 2018).

### 2.4 Microbial diversity, composition, and taxonomic differential relative abundance

To test the stability or variability of the microbiome over time, comparisons were made under three conditions: J‐20, S‐20, and M‐21. To determine microbial diversity among the conditions, alpha and beta diversity metrics were calculated using q2‐diversity plugin in QIIME 2 (Bolyen et al., 2019). Three alpha diversity metrics were explored using observed features (DeSantis et al., 2006) and Faith's phylogenetic diversity index (Faith, 1992) for richness, while evenness was explored with the Pielou's evenness index (Pielou, 1966). Differences in alpha‐diversity metrics between groups were assessed with the Kruskal–Wallis test (*p* ≤ .05) using QIIME 2 (Bolyen et al., 2019). Beta‐diversity was assessed with the Bray–Curtis dissimilarity index (Bray & Curtis, 1957) with the PERMANOVA test (*p* ≤ .01) on QIIME 2. Beta dispersion was calculated using the betadisper function and the Vegan script implemented in RStudio (Oksanen et al., 2021), using an ANOVA test (*p* ≤ .05) as statistical analyses. Cluster analysis was performed with the Jaccard coefficient of similarity using Vegan (Oksanen et al., 2021) implemented in RStudio (RStudio Team, 2020). Unique and shared taxa among the three conditions were represented using Venn diagrams created with an online tool (http://bioinformatics.psb.ugent.be/webtools/Venn/).

Differences in taxa relative abundance between the three conditions were tested using a Kruskal–Wallis test (*p* ≤ .05) and implemented using the ANOVA‐Like Differential Expression (ALDEx2) package (Fernandes et al., 2013) on RStudio (RStudio Team, 2020). Only taxa with significant differences (*p* ≤ .05) were used for representation of the differential taxa relative abundance. Relative abundance was measured as centred log ratio (clr) transformation. The identified differentially abundant taxa were used to create a heatmap using the package “Heatplus” in RStudio (RStudio Team, 2020).

### 2.5 Inference of bacterial co‐occurrence networks

Co‐occurrence networks were created for each dataset using the taxonomic profiles at family and genera level. The networks provide a graphical representation of the assembly of complex microbial communities within ecosystems. It allows us to analyze the associations that are established, as well as their nature. In the assembly, the nodes represent the taxa, while the edges represent the associations established between them. Analyses of significant positive (weight > 0.75) or negative (weight < −0.75) correlations were performed using the Sparse Correlations for Compositional data (SparCC) method (Friedman & Alm, 2012), implemented in RStudio (RStudio Team, 2020). Visualization and measurement of topological features (i.e., number of nodes and edges, network diameter, modularity, average degree, weighted degree, clustering coefficient and total count of basic undirected motifs of three fully connected vertices [triangles]) of the networks were performed using Gephi v0.10 (Bastian et al., 2009).

With the aim of identifying microbial taxa shared for the conditions, a Core Association Networks (CAN) were created for J‐20/S20, S20/M21, and J‐20/M21, using a software toolbox, anuran (a toolbox with null models for identification of nonrandom patterns in association networks) (Röttjers et al., 2021), and this version was tested in Python 3.6.

### 2.6 Keystone taxa identification

Keystone taxa were identified within the community for each of the condition, based on three criteria, as previously reported (Mateos‐Hernández et al., 2021): (i) ubiquitousness (microbial taxa present in all samples in an experimental group), (ii) eigenvector centrality higher than 0.75, and (iii) high mean relative abundance (i.e., higher than that of the mean relative abundance of all taxa in an experimental group). Additionally, the common keystone taxa for J‐20, S‐20, and M‐21 microbial community were identified.

### 2.7 Local connectivity of *Anaplasma* in the microbial community

To explore the role of *Anaplasma* within the community, its direct relationship with the rest of the bacterial microbiome was determined. For this purpose, subnetworks were constructed where *Anaplasma* was visualized with its direct positive and negative associations. The analyses were carried out in Gephi v0.10 (Bastian et al., 2009), and the strength of the edges was presented with the SparCC weight.

### 2.8 Analysis of centrality measures distribution in network nodes

The topology of the taxa in the network was analyzed with two connectivity types: (i) within‐module connectivity (*Z*_i_), which describes how the taxon is connected to others within its module and (ii) among‐module connectivity (*P*_i_), which describes the taxa connectivity with other taxa in different modules (Guimera & Nunes Amaral, 2005). The taxa are divided into four categories: (i) peripherals taxa (*Z*_i_ ≤ 2.5 and *P*_i_ ≤ 0.62), which contain taxa with few edges in and out of its module; (ii) connectors (*Z*_i_ ≤ 2.5 and *P*_i_ > 0.62), which contain taxa connected to other modules than its own; (iii) module hubs (*Z*_i_ > 2.5 and *P*_i_ ≤ 0.62), which contain taxa highly connected with members of their own module; and (iv) network hubs (*Z*_i_ > 2.5 and *P*_i_ > 0.62), which contain taxa highly connected with members within and among its module. For each taxon, *Z*_i_ and *P*_i_ values were calculated using only positive edges, with the R package “code‐zi‐pi‐plot” described by (Cao et al., 2018) and (Guo et al., 2022) in Rstudio (R studio Team, 2020) and visualized with GraphPad Prism version 8.0.1 (GraphPad Software, San Diego, California USA).

### 2.9 Differential network analysis and modules composition

With the aim of comparing the correlations between the same taxa in two different bacterial networks, a statistical network estimation analysis was performed using the network construction and comparison for microbiome (NetCoMi) method (Peschel et al., 2021) implemented in RStudio (RStudio Team, 2020). The comparison was carried out: (i) with *Anaplasma* (wA) and (ii) without *Anaplasma* (woA) (*in*‐*silico* removal) for each time point, (iii) wA vs. woA in the same time point. To test for dissimilarities between the two networks [i.e., J‐20 vs. S‐20; J‐20 (wA) vs. J‐20 (woA)], the Jaccard index was calculated to test for dissimilarities between nodes in the two networks for degree, betweenness centrality, closeness centrality and eigenvector centrality. The Jaccard index tests for the similarity between sets of “most central nodes” of networks, which are defined as those nodes with a centrality value above the empirical 75% quartile. This index expresses the similarity of the sets of most central nodes as well as the sets of hub taxa between the two networks. The Jaccard index ranges from 0 (completely different sets) to 1 (sets equal). The two *p*‐values *p* (*J* ≤ *j*) and *p* (*J* ≥ *j*) for each Jaccard index are the probability that the observed value of Jaccard's index is “less than or equal” or “higher than or equal,” respectively, to the Jaccard value expected at random which is calculated taking into account the present total number of taxa in both sets (Real & Vargas, 1996). The ARI was calculated to test the dissimilarity of clustering in the networks. The ARI values range from −1 to 1. Negative and positive ARI values mean lower and higher than random clustering, respectively. An ARI value of 1 corresponds to identical clustering and 0 to dissimilar clustering. The *p*‐value tests whether the calculated value is significantly different from zero (Peschel et al., 2021).

To assess the potential direct and indirect consequences of removing *Anaplasma* from the networks, we focused on two modules (M1 and M2) within the networks. M1 represented the module containing *Anaplasma*, while M2 exhibited a higher number of taxa and modularity value. This ensured the equivalence of modules between wA and woA networks. Subsequently, subnetworks were constructed for comparison (wA vs. woA) at the same time point, to better understand the network dynamics.

### 2.10 Network robustness analysis in nodes removal and addition

The robustness of the networks against disturbances due to removal and addition of nodes was determined. In the analysis of node removal, the proportion of eliminated nodes necessary to achieve a connectivity loss of 0.40 (40%) and 0.80 (80%) was recorded, after directed and random attacks. Two scenarios were evaluated: (i) robustness of the networks at each time point (J‐20, S‐20 and M‐21) and (ii) robustness of the (wA‐woA) networks at the same time point. The robustness of the networks was calculated using the Network Strengths and Weaknesses Analysis (NetSwan) package (Lhomme, 2015) in RStudio (RStudio Team, 2020).

The robustness of the networks at each time point (J‐20, S‐20, and M‐21) and at the same time point (wA vs. woA) was analyzed for the addition of nodes, using the Network analysis and visualization package (Freitas et al., 2021). Nodes were incrementally added in sections ranging from 5 to 100, and network connectivity was measured based on the degree metric of the largest connected component (LCC) and average path length. A Wilcoxon signed‐rank test was conducted to calculate *p*‐values for LCC and average path length. The *p*‐values were adjusted using the Benjamini–Hochberg (BH) method to control the false discovery rate. Additionally, bootstrapping was performed to obtain confidence intervals for the variables. Significance was determined at a threshold of *p* < .05.

## 3 RESULTS

### 3.1 Diversity, composition, and abundance of bacterial taxa in *R. microplus* microbiome over time

**FIGURE 1:**
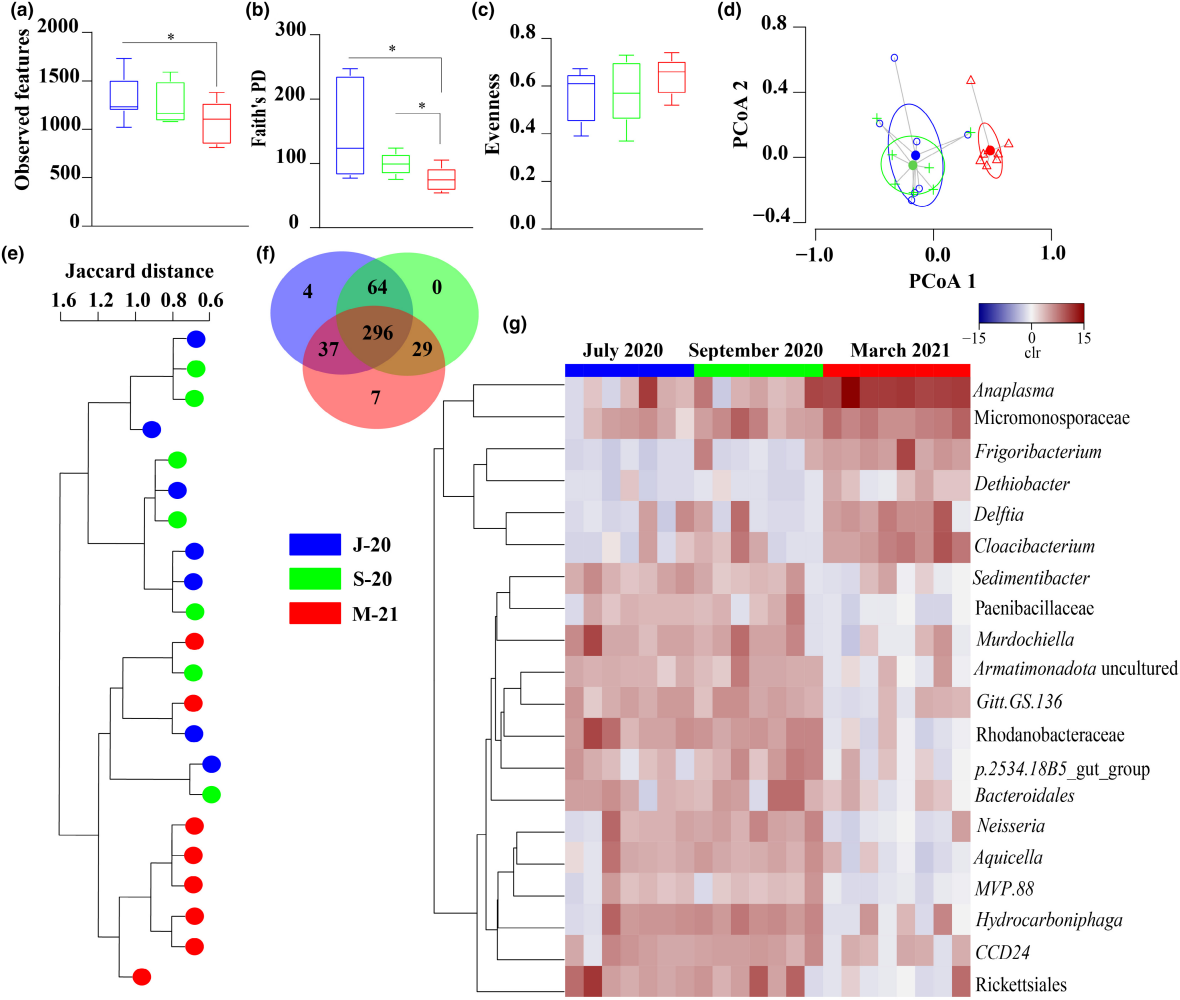
Comparison of diversity of complex microbial communities within *Rhipicephalus microplus* over time. Comparison of alpha diversity between J‐20, S‐20 and M‐21 (Kruskal–Wallis test, significant differences for *p* ≤ .05), (a) observed features, (b) Faith's phylogenetic diversity (PD), and (c) Pielou's evenness index. (d) Comparison of beta – diversity with Bray Curtis dissimilarity index between J‐20, S‐20, and M‐21. Beta dispersion of three sets of samples (global comparison). Small circles, crosses and triangles represent samples, and ellipses represent centroid position for each group. This test use principal coordinate analysis (PCoA), it is used to explore and to visualize variability in a microbial community. ANOVA test was performed and showed that beta dispersion of the three sets of samples (three conditions) is not significantly different (*p* = .51). (e) Jaccard clusterisation of the tick samples collected in J‐20, S‐20, and M‐21. The samples are represented by circles and the groups by colors (legend). (f) Venn Diagram displaying the comparison of taxa composition in ticks collected at the three sampling times. Common and unique taxa between the conditions are represented. (g) Comparison of relative abundance of complex microbial communities within *R. microplus* over time. The taxa were clustered based on relative abundance (calculated as clr transformed values). Each column represents the clr values for bacterial taxa per sample and per group. Each line represents bacterial taxa with significant changes between the datasets. Color represent the clr value (range from −15 to 15).

Diversity, composition, and abundance of bacterial taxa in the *R. microplus* microbiome were assessed over two consecutive years, 2020 and 2021, using 16S rRNA gene profiling after statistical identification and removal of DNA features identified as contaminants (Table S2). Differences in α‐diversity were significant, with higher observed features in ticks collected in J‐20 compared to those collected in M‐21 (Kruskal–Wallis, *p* = .049, Figure 1a). Faith's phylogenetic diversity (Faith's PD) also differed between M‐21 and J‐20 (Kruskal–Wallis, *p* = .015, Figure 1b), as well as M‐21 and S‐20 (Kruskal–Wallis, *p* = .021, Figure 1b). However, no significant differences were observed between J‐20 and S‐20 for observed features (Kruskal–Wallis, *p* > .05, Figure 1a) or Faith's PD (Kruskal‐Wallis, *p* > .05, Figure 1b) metrics. Evenness showed no significant changes over time (*p* > .05, Figure 1c).

Bray–Curtis index analysis revealed no significant differences in microbiome composition between J‐20 and S‐20 (PERMANOVA, *p* > .01), but both differed from M‐21 (PERMANOVA, *p* = .001, *F* = 2.652). Beta dispersion showed no significant within‐group variability (ANOVA test, *p* > .05, Figure 1d). Jaccard clustering confirmed two distinct clusters: one with all M‐21 samples and two with J‐20 and S‐20 samples (Figure 1e), aligning with Bray–Curtis index findings. Compositional analysis identified 437 bacterial taxa, with 67.7% shared across all samples (Figure 1f). Unique taxa were found in M‐21 (1.60%) and J‐20 (0.92%), while S‐20 had none (Figure 1f, Table S3).

Differential relative abundance analysis identified significant changes in 20 taxa across the three conditions (Figure 1g, Table S4). *Murdochiella*, *Neisseria*, and Rickettsiales were more abundant in J‐20 and S‐20, while *Anaplasma*, *Cloacibacterium*, *Delftia*, and *Frigoribacterium* were higher in M‐21. *Anaplasma* consistently showed significantly higher mean relative abundance in M‐21 (10.8 ± 1.41) compared to J‐20 (3.01 ± 0.78) and S‐20 (4.36 ± 0.93) (*p* < .05), aligning with previous PCR‐confirmed findings (Piloto‐Sardiñas, Foucault‐Simonin, et al., 2023). The increased relative abundance of *Anaplasma*, coupled with reduced microbial diversity over time, suggest a potential interaction with *R. microplus* microbial communities.

### 3.2 Dynamics of *Anaplasma* nesting in the microbial communities of *R. microplus*

**FIGURE 2:**
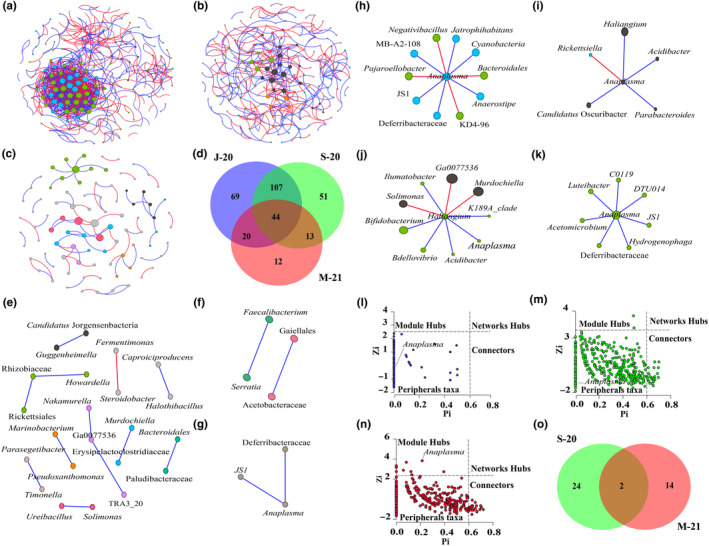
Dynamics of *Anaplasma* nesting in the microbial communities of *Rhipicephalus microplus* over time. Global and local co‐occurrence networks. Co‐occurrence networks of (a) J‐20, (b) S‐20, and (c) M‐21 networks. Node colors are based on modularity class metric and equal color means modules of co‐occurring taxa. The size of the nodes is proportional to the eigenvector centrality of each taxon. The colors in the edges represent strong positive (blue) or negative (red) correlations (SparCC >0.75 or <−0.75). (d) Venn diagram displaying the comparison of networks composition. CAN for: (e) J‐20/ S‐20, (f) S‐20/M‐21, and (g) J‐20/M21, the colors in the edges represent strong positive (blue) or negative (red) correlations and the nodes represent bacterial taxa. Sub‐networks of local connectivity and indirect association of *Anaplasma* with keystone bacteria within the bacterial community in *R. microplus* over time: *Anaplasma*'s local connectivity within (h) J‐20 and (i) S‐20 networks, (j) *Anaplasma*'s indirect association with keystone bacteria in S‐20 network and (k) *Anaplasma*'s local connectivity within M‐21 network. Within‐module and among‐module connectivities, *Z*_i_‐*P*_i_ plot of the individual genera from three groups: (l) J‐20, (m) S‐20, and (n) M‐21. (o) Venn diagram displaying the comparison of the connectors between the S‐20 and M‐21 networks.

The dynamics of *Anaplasma* nesting within the microbial communities of *R. microplus* were investigated using co‐occurrence networks to assess community assembly over time. Notably, J‐20 displayed the most total and connected nodes, while M‐21 had the least, showcasing topological variations (Figure 2a–c, Table 1). Additionally, J‐20 displayed the highest number of correlations with a balanced positive–negative ratio (Figure 2a, Table 1). In contrast, S‐20 and M‐21 showed greater differences in positive–negative associations (Figure 2b,c, Table 1). Despite M‐21 having fewer connected nodes, it had a high proportion of positive associations (Figure 2c, Table 1). S‐20 and M‐21 displayed lower modularity than J‐20. The three networks had similar diameter values between them (Table 1). The total count of motifs over time was determined (Table 1). The J‐20 network presented the highest total number of motifs (13,322), followed by S‐20 (519), while M‐21 (18) had the lowest (Table 1). The high modularity values and a considerable number of motifs within J‐20 (Table 1) indicate a strong connection between the vertices (nodes) compared to the S‐20 and M‐21 networks (Table S5). Compositional analysis revealed that J‐20 had the higher number of unique nodes (69), followed by S‐20 (51) and M‐21 (12) (Figure 2d, Table S6). The significant overlap in nodes between J‐20 and S‐20 compared to M‐21 implies minimal taxa variability and minimal assembly variation between J‐20 and S‐20 (Figure 2a–d).

**TABLE 1:** Topological features of J‐20, S‐20, and M‐21 networks with (wA) and without (woA) *Anaplasma* for each time.

Topological features	J‐20 (wA)	S‐20 (wA)	M‐21 (wA)	J‐20 (woA)	S‐20 (woA)	M‐21 (woA)
Total nodes	401	389	369	400	388	368
Connected nodes	240	215	90	231	195	91
Edges	975	369	76	867	375	72
Positive correlations	514 (52.7%)	232 (62.9%)	48 (60.5%)	493 (56.9%)	222 (59.2%)	44 (61.1%)
Negative correlations	461 (47.3%)	137 (37.1%)	28 (36.8%)	374 (43.1%)	153 (40.8%)	28 (38.9%)
Modularity	6.43	2.09	2.01	3.24	2.61	2.46
Network diameter	12	15	10	12	14	14
Average degree	8.13	3.43	1.69	7.5	3.85	1.58
Weighted degree	0.40	0.71	0.36	0.86	0.57	0.27
Clustering coefficient	0.52	0.39	0.29	0.46	0.38	0.21
Number of motifs	13,322	519	18	9549	561	12

The core association network (CAN) revealed 22 core associated nodes between J‐20 and S‐20 networks, supporting the observation of minimal variability in the assembly of the microbial community in these two conditions (Figure 2e). In contrast, four and three core associated nodes were found between M‐21 and S‐20 (Figure 2f), and J‐20 networks (Figure 2g), respectively. Interestingly, *Anaplasma* was absent in the cores shared by J‐20/S‐20 and J‐20/M‐21 (Figure 2e,f), but present in the S‐20/M‐21 core, establishing a positive association with *JS1* (Caldatribacteriota) and Deferribacteraceae (Figure 2g). The presence of *Anaplasma* as a core associated node suggests that, alongside its increased abundance in the community, this pathogen may acquire importance in the community across time.

Analyzing the local connectivity of *Anaplasma* across the three specified time points revealed dynamic relationships. In the J‐20 network, *Anaplasma* displayed numerous direct associations with nodes exhibiting high eigenvector centrality values (*Pajaroellobacter*, *KD4‐96*, *Bacteroidales*, Deferribacteraceae) compared to other nodes in the network (Figure 2h). However, despite this, their mean clr values were lower than the mean relative abundance of all taxa in the experimental condition, disqualifying them as keystone taxa. In the S‐20 network, *Anaplasma* mostly exhibited positive associations, except for a negative association with *Rickettsiella* (Figure 2i). Notably, in this condition, *Anaplasma* had higher eigenvector centrality compared to J‐20, with a mean relative abundance value below the average relative abundance of all taxa in S‐20. This node indirectly linked with three keystone taxa (Ga0077536, *Murdochiella*, *Solimonas*) (Table 2), through a direct association with *Haliangium* (Figure 2j). Notably, in the M‐21 network, *Anaplasma* appeared as a keystone taxon (Table 2), with positive association module with seven nodes (Figure 2k). In the M‐21 network, *Rickettsiella* was no longer part of the network, possibly due to co‐exclusion caused by a negative interaction observed with *Anaplasma* in S20 (Figure 2i). Most direct associations of *Anaplasma* at a given time point were either displaced or did not co‐occur with the taxon in the rest of the conditions, except for Deferribacteraceae and *JS1* (Caldatribacteriota) in J‐20 and M‐21 (Table S7).

**TABLE 2:** Keystone taxa of the bacterial communities within *R. microplus* by condition and in common.

Condition	Keystone taxa by condition and in common
July 2020	TRA3‐20 *Flavonifractor* Rhizobiaceae uncultured *Guggenheimella* *Hydrocarboniphaga* Ga0077536 *Ideonella* *Nakamurella* *Acidibacter* Staphylococcaceae *Parvibacter* *Aquicella*
September 2020	Ga0077536 *Murdochiella* Acetobacteraceae *Solimonas*
March 2021	*Quadrisphaera* *Acidibacter* *Anaplasma* Cellulomonadaceae
July 2020–September 2020	Ga0077536
July 2020–March 2021	*Acidibacter*

The evaluation of within‐module (*Z*_i_) and among‐module (*P*_i_) connectivity revealed similar distributions of nodes across the three conditions, with most nodes as peripheral (low *Z*_i_ and *P*_i_) and no network hubs identified (high *Z*_i_ and *P*_i_; Figure 2l–n). However, the presence of module hubs differed among the three groups, with nine, two and none taxa considered as module hubs in M‐21, S‐20, and J‐20 networks, respectively (Figure 2l–n). The analysis revealed 24 and 14 unique connectors in S‐20 and M‐21 networks, respectively. Propionibacteriaceae and Lachnospiraceae UCG‐002 promote coherence of both networks (Figure 2o, Table S8). *Anaplasma*'s position within the networks varied; it was peripheral in J‐20 and S‐20 networks (Figure 2l,m), but contributed to module coherence in the M‐21 network (Figure 2n).

*Anaplasma* exhibited varying associations, evolving from diverse connections in J‐20 to higher centrality and positive associations in S‐20. Remarkably, in M‐21, *Anaplasma* emerged as a keystone taxon, forming an independent cluster nested in the network establishing positives associations with other taxa. The absence of certain taxa and the potential co‐exclusion of *Rickettsiella* may underscore the distinctive impact of *Anaplasma* on the community's dynamics over time.

### 3.3 Influence of *Anaplasma* on the assembly, and hierarchy of *R. microplus* microbiome over time

To investigate *Anaplasma*'s impact on community assembly, network topology after its removal (woA) was analyzed and compared to the network with *Anaplasma* (wA). The removal of *Anaplasma* led to the loss of nodes in both the J‐20 (woA) and S‐20 (woA) networks, resulting in the depletion of both positive and negative correlations (Table 1, Figure S1A,B). Notably, within S‐20 (woA) network, the associations changed with 16 new negative correlations appearing and 10 positive correlations being lost, suggesting that *Anaplasma* exerts an influence on interactions within the microbial community (Table 1, Figure S1B). In M‐21 (woA), only one new connection formed within the network, and four positive correlations were lost (Table 1, Figure S1C). The removal of *Anaplasma* resulted in a decrease in the total number of motifs across all three networks, with the modularity value in the J‐20 (woA) network halving (Table 1). However, despite the removal of *Anaplasma*, the J‐20 (woA) network retained a substantial number of motifs compared to the S‐20 (woA) and M‐21 (woA) networks, suggesting that association patterns among taxa with shared functions evolve over time (Table 1, Table S5). Overall, the removal of *Anaplasma* did not lead to significant changes in other topological features (Table 1). Regarding the composition, 53 nodes were shared by the three networks (Figure S1D, Table S9). J‐20 (woA) network had the most unique nodes, followed by S‐20 (woA) and M‐21 (woA) (Figure S1D, Table S9).

Jaccard index comparison was used as a test to evaluate the local centrality measures in networks. The analysis considered dynamic interaction patterns within (wA and woA) networks at each time point and evaluated the impact of *Anaplasma*'s removal (wA vs. woA) at the same time point. Jaccard index values of the comparisons of centrality measures, were lower than expected by random in the wA and woA network comparison for each time point (*p* (≤ Jacc) < .05). With the exception, in both cases, of betweenness centrality in the J/S‐20 comparison which has a random distribution (Tables S10 and S11). On the other hand, when the Jaccard index values were compared, the centrality measures between the (wA vs. woA) networks, in the same time point, were higher than expected by random (Table S12). The comparison of node clustering for wA and woA networks, in each time point, showed the higher ARI value for J‐20 compared to S‐20 networks, followed by S‐20/M‐21. The J‐20/M‐21 comparisons showed low similarities in clustering (Table 3). When both, the dynamic interaction patterns and impact of *Anaplasma*'s removal were analyzed, the high dissimilarity between J‐20 and M‐21 is in correspondence with what was observed visually and by the topological features values. When comparing wA vs. woA networks at the same time point, we found a higher ARI value, close to 1, indicating strong similarities and suggesting that *Anaplasma*'s removal did not affect clustering (Table 3).

**TABLE 3:** Network clustering comparisons.

Conditions	Network comparisons	Adjusted Rand index (ARI)	*p*‐Value
With *Anaplasma* (wA)	J‐20 (wA) vs. S‐20 (wA)	0.25	0***
	S‐20 (wA) vs. M‐21 (wA)	0.14	0***
	J‐20 (wA) vs. M‐21 (wA)	0.09	0***
Without *Anaplasma* (woA)	J‐20 (woA) vs. S‐20 (woA)	0.26	0***
	S‐20 (woA) vs. M‐21 (woA)	0.18	0***
	J‐20 (woA) vs. M‐21 (woA)	0.09	0***
wA vs. woA	J‐20 (wA vs. woA)	0.82	0***
	S‐20 (wA vs. woA)	0.81	0***
	M‐21 (wA vs. woA)	0.79	0***

**FIGURE 3:**
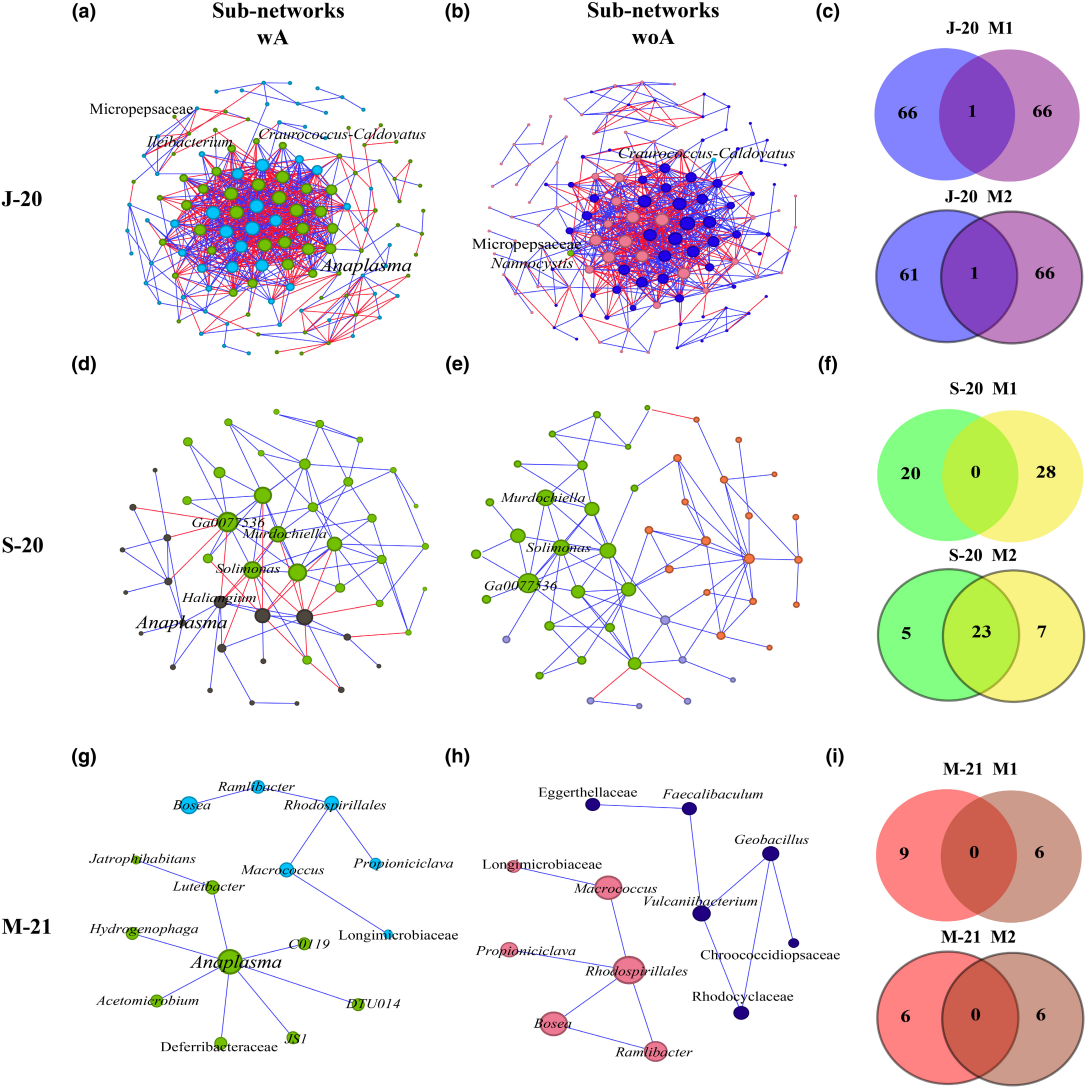
Co‐occurrence networks of the main modules (M1 and M2). Sub‐networks that contain M1 and M2 from global co‐occurrence networks in *Anaplasma*'s presence (wA) and removal (woA). Venn diagram displaying the comparison of module composition (M1‐M2) in *Anaplasma*'s presence and removal: (a) J‐20 wA, (b) J‐20 woA, (c) J‐20 (M1M2), (d) S‐20 wA, (e) S‐20 woA, (f) S‐20 (M1M2), (g) M‐21 wA, (h) M‐21 woA, and (i) M‐21 (M1M2).

The impact of removing *Anaplasma* was evaluated on two modules: M1, where *Anaplasma* was initially present, and M2, a module with a higher number of taxa and modularity value indirectly affected by *Anaplasma*'s removal. Micropepsaceae and *Craurococcus*‐*Caldovatus* were the only common taxa found in the comparison between the J‐20 (wA vs. woA) networks in modules 1 (J‐20 M1) and 2 (J‐20 M2), respectively (Figure 3a,b). After *Anaplasma*'s removal, both the composition of the modules and the direct associations of the shared taxa changed drastically (Figure 3b,c, Table S13). In the S‐20 network, *Anaplasma* is contained in the M1 in indirect association through *Haliagium* with keystone taxa found in M2 (Figure 3d). After *Anaplasma*'s removal, the composition of the M1 changes drastically, no taxon is shared (Figure 3e,f). Keystone taxa's presence in M2 (wA vs. woA) contributes to the similarity in the composition of the module, sharing a total of 23 taxa (Figure 3d–f, Table S13). As a result of the large number of shared nodes in M2 (wA vs. woA), due to the stability in the composition conferred by the presence of keystone taxa, the majority of the connected nodes represented in the differential sub‐network correspond to M1 (wA and woA) (Figure 3f). After the analysis of both modules in the M‐21 network, no taxon was shared, which may be due to the independent clustering formed by *Anaplasma* and its role as keystone taxa (Figure 3g–i, Table S13).

### 3.4 Influence of *Anaplasma* on network robustness

A comprehensive analysis of network stability and capacity to withstand disturbances, such as node removal and addition, was performed over different months, both in the presence and absence of *Anaplasma* at the same time point. The robustness of networks with *Anaplasma* against directed and random attacks was assessed by comparing the fraction of nodes necessary for a connectivity loss of 40% and 80% over time.

**FIGURE 4:**
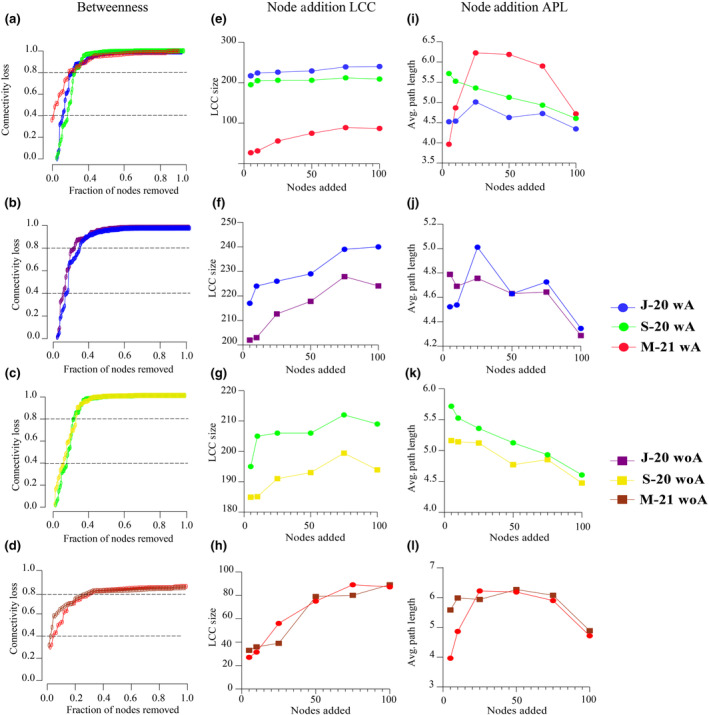
Robustness comparison between J‐20, S‐20, and M‐21 networks after removal and addition of nodes in *Anaplasma*'s presence and removal. (a) Connectivity loss measured after directed attack in *Anaplasma*'s presence between networks for each time point, removing first the nodes with the highest betweenness centrality (J‐20 wA/S‐20 wA/M‐21 wA). Connectivity loss measured after directed attack in *Anaplasma*'s presence and removal between networks in the same point time, removing first the nodes with the highest betweenness centrality: (b) J‐20 (wA vs. woA), (c) S‐20 (wA vs. woA), (d) M‐21 (wA vs. woA). Largest connected component (LCC) values are represented and compared in *Anaplasma*'s presence between networks for each time point: (e) J‐20 (wA)/S‐20 (wA)/M‐21 (wA). LCC values are represented and compared in *Anaplasma*'s presence and removal between networks in the same point time: (f) J‐20 (wA vs. woA), (g) S‐20 (wA vs. woA), and (h) M‐21 (wA vs. woA). Average path length (APL) values are represented and compared in *Anaplasma*'s presence between networks for each time point: (i) J‐20 (wA)/S‐20 (wA)/M‐21 (wA). APL values are represented and compared in *Anaplasma*'s presence and removal between networks in the same point time: (j) J‐20 (wA vs. woA), (k) S‐20 (wA vs. woA), and (l) M‐21 (wA vs. woA).

Results indicated that J‐20 (wA) and S‐20 (wA) network connectivity remained similar after directed attacks in betweenness (Figure 4a), cascading (Figure S1E), degree (Figure S1F), as well as random attack (Figure S1G), removing equal fractions of nodes caused losses of connectivity of 40% and 80% (Table S14). This behavior corresponded with the random distribution for betweenness centrality observed in the comparison of local centrality measures (Table S10). In contrast, the M‐21 network was severely affected during directed and random attacks due to its lower number of nodes and motifs compared to J‐20 and S‐20 (Figure 4a, Figure S1E–G, Table S14).

As demonstrated, *Anaplasma* influenced community assembly by slightly modifying topological features and drastically changing the composition, especially in modules where it was present. *Anaplasma*'s removal did not alter the robustness of J‐20 woA and S‐20 woA networks against directed (cascading and degree) and random attacks (Figure S1H–M, Table S14). However, the M‐21 woA network experienced a rapid loss of connectivity (80%) compared to M‐21 wA at a lower fraction of removed nodes, against both directed and random attacks (Figure S1N–P, Table S14). The most notable robustness behavior occurred when comparing networks in the *Anaplasma*'s presence and removal after a directed attack in betweenness (Figure 4b–d). In J‐20 and S‐20, *Anaplasma*'s removal did not significantly change the proportion of nodes required for a loss of connectivity (Figure 4b,c, Table S14). Conversely, in M‐21, *Anaplasma*'s removal conferred instability to the networks, making them more susceptible to disturbances (Figure 4b,c, Table S14).

As a result of the addition of nodes, the values of LCC Size and average path length were compared between the networks at each time point (J‐20, S‐20 and M‐21) and at the same time point (wA vs. woA) (Figure 4e–l, Table S15). In general, J‐20 and S‐20 networks maintained similar and larger LCC Size values compared to M‐21 network (Figure 4e, Table S15). The wA networks maintained larger values of LCC size compared to the woA networks in J‐20 (Figure 4f, Table S15) and S‐20 (Figure 4g, Table S15). In contrast, the M‐21 (wA vs. woA) networks the LCC size values were more similar with overlap at some points (Figure 4h, Table S15). However, in all cases, the value of LCC Size was increased as greater number of nodes were added. With respect to the average path length J‐20 and S‐20 networks maintained similar values and behavior compared to M‐21 (Figure 4i, Table S15). Examining the (wA vs. woA) APL comparison, the similarity and overlapping of the values at some points between the networks in the presence and absence of *Anaplasma* was evident in J‐20 and M‐21, while in S‐20 they were different and approached as the number of nodes added increased (Figure 4j–l, Table S15). This predictive behavior, in the conditions where *Anaplasma* is important in the community either due to abundance, direct association with key taxon or its own role as a keystone, suggests that its presence confers stability in the assembly of the microbial community in the face of a possible loss or addition of taxa.

## 4 DISCUSSION

The dynamic nature of microbial communities within ticks is a topic of considerable interest due to its potential implications for tick‐borne diseases. In this study, we aimed to elucidate the stable and variable elements in the *R. microplus* microbiome over time and explore the interactions between microbial communities and specific pathogens, with a focus on *Anaplasma* nesting within these communities over time. The tick microbiome is considered a fluctuating microecosystem influenced by internal factors, such as interactions among pathogenic and nonpathogenic microorganisms, and responses to external perturbations (Aguilar‐Díaz et al., 2021; Cabezas‐Cruz et al., 2018; Chicana et al., 2019; Swei & Kwan, 2017; Wu‐Chuang et al., 2021). To analyze the interplay between pathogens and the temporal dynamics of microbial communities: the covariates (engorgement level, tick species, stages and host) were reduced, individual tick samples positive for single *A. marginale* infection were selected, and extraction process controls were used.

Observations revealed differences in α‐diversity between ticks collected in different years and a significantly higher relative abundance of *Anaplasma* in ticks from M‐21 compared to J‐20 and S‐20, indicating substantial temporal variation in the *R. microplus* microbiome. Changes in microbial diversity demonstrated that the tick microbiome is a dynamic system with varying patterns of species abundance, suggesting interference in the acquisition and colonization process in *R. microplus* due to *Anaplasma*'s relative abundance variability. High microbial diversity within ecosystems makes communities more resistant to pathogen colonization, as trophic interactions prevent dominance by a single pathogen (Wei et al., 2015). Although differences in the abundance of bacterial taxa in the presence of *Anaplasma* imply its impact on the *R. microplus* microbiome, further exploration is necessary to understand the co‐occurrence patterns it establishes with the bacterial community over time.

The microbial community assembly in *R. microplus* reveals a dynamic interplay among microorganisms, forming intricate and ever‐changing microbial consortia. The configuration of microbial communities in ecosystems is likely influenced by various biotic interactions, such as commensalism, mutualism, and parasitism, among the composing microorganisms (Freilich et al., 2011; Nemergut et al., 2013; Stolyar et al., 2007). Comparative analyses of bacterial assembly in hard ticks (Maitre et al., 2023) and soft ticks (Piloto‐Sardiñas, Cano‐Argüelles, et al., 2023) indicate a higher frequency of positive interactions between bacterial taxa. Our co‐occurrence pattern analysis in *R. microplus* underscores a predominant occurrence of cooperative interactions across all three conditions. However, a notable decline in associations among community members was evident over time.

The nature of microbial cooperation or competition hinges on factors such as metabolic diversity (Stolyar et al., 2007), genotypic and phenotypic variations between species (Ackermann, 2015; Mitri & Richard Foster, 2013), environmental carrying capacity (Freilich et al., 2011) and the presence, role and microbial load of pathogenic microorganisms (Adegoke et al., 2020; Maitre et al., 2022). Consequently, the observed reduction in associations across the three conditions may be attributed to the loss of bacterial taxa or the increasing influence of *Anaplasma* within the community over time.

Over time, assembly patterns in the microbial community have evolved, marked by the emergence of *Anaplasma* as a key taxon. *Anaplasma* forms an independent cluster within the M‐21 network, while there is a notable reduction in bacterial taxa, with *Rickettsiella* being one of the taxa displaced from the community. Previous investigations *in I. ricinus* ticks indicated no preferential or antagonistic association between *Rickettsiella* and *Borrelia* species (*B. burgdorferi* and *B. miyamotoi*) (Garcia‐Vozmediano et al., 2022). In contrast, our network analysis suggests a potential displacement of *Rickettsiella* by *Anaplasma*, possibly due to competition or co‐exclusion interactions established between the two in the preceding condition. The variations in co‐occurrence and nesting patterns of *Anaplasma* at the three time points, along with its presumed impact on the reduction of bacterial taxa, indicate a critical role played by this pathogen in shaping interactions within the microbial community and influencing the dynamics of the *R. microplus* microbiome.

*In silico* removal of *Anaplasma* reveals modified interaction patterns, motif numbers, module compositions, and stability against disturbances across time points. The most significant impacts are observed when *Anaplasma* establishes direct interaction with keystone taxa or is considered one. Recent evidence indicates that bacterial pathogens like *R. helvetica* (Maitre et al., 2022), and *B. afzelii* (Wu‐Chuang et al., 2023) modulates the composition and assembly of the bacterial community in *I. ricinus*, while *A. phagocytophilum* modifies the *I. scapularis* microbiome thus facilitating its colonization (Abraham et al., 2017).

Studies also show that the removal of *Rickettsia* affects conserved patterns of community assembly in *H. marginatum* and *R. bursa* ticks, suggesting it acts as a community assembly driver (Maitre et al., 2023). Conversely, in *R. microplus*, infection by the protozoan *Theileria* sp. leads to a significant reduction in the bacterial community, termed “pathogen‐induced dysbiosis” by the authors (Adegoke et al., 2020). Keystone taxa, in general, influence the composition, structure, assembly, and functioning of microbial communities (Banerjee et al., 2018; Modlmeier et al., 2014). Those sustaining and stabilizing a microbiome associated with pathological states are referred to as “keystone pathogens” (Hajishengallis et al., 2012). When these pathogens dominate the community by suppressing other microbes, they induce an alteration in the microbiome (Hajishengallis et al., 2012).

The changes in microbial community assembly induced by *Anaplasma* and its role as a keystone taxon suggest a “keystone pathogen‐induced dysbiosis” in *R. microplus*. Although further studies are required to validate this hypothesis, we propose that the effects induced by *Anaplasma* on the community dynamics likely create a favorable environment facilitating its colonization within *R. microplus*.

In summary, our findings indicate that the microbiome of *R. microplus* is a dynamic system. The results highlight the significant influence of *A. marginale* on microbial communities, suggesting that its high relative abundance, widespread presence, and increasing importance shape the dynamics of microbial interactions. This, in turn, may potentially alter the physiology and vector capacity of *R. microplus*. Analyzing microbiome dynamics is crucial for understanding the cause‐and‐effect relationships in responses to both biotic and abiotic factors. Recognizing the temporal dimension in tick–microbiome interactions becomes fundamental for strategies aiming to manipulate bacterial communities to modify tick physiology and vector capacity. Nevertheless, further research is required to uncover the mechanistic basis of these effects and their broader implications for the transmission dynamics of tick‐borne pathogens, the ecology of tick microbiome, and the development of effective control strategies.

## AUTHOR CONTRIBUTIONS

**Elianne Piloto‐Sardiñas:** Conceptualization (equal); formal analysis (lead); investigation (lead); validation (lead); visualization (lead); writing – original draft (lead); writing – review and editing (equal). **Lianet Abuin‐Denis:** Formal analysis (supporting); writing – review and editing (equal). **Apolline Maitre:** Formal analysis (supporting); supervision (supporting); writing – review and editing (equal). **Angélique Foucault‐Simonin:** Investigation (supporting); writing – review and editing (equal). **Belkis Corona‐González:** Conceptualization (equal); investigation (supporting); resources (equal); supervision (equal); writing – review and editing (equal). **Cristian Díaz‐Corona:** Writing – review and editing (equal). **Lisset Roblejo‐Arias:** Writing – review and editing (equal). **Lourdes Mateos‐Hernández:** Formal analysis (lead); writing – review and editing (equal). **Roxana Marrero‐Perera:** Investigation (equal); writing – review and editing (supporting). **Dasiel Obregon:** Software (lead); writing – review and editing (equal). **Karolína Svobodová:** Formal analysis (equal); methodology (equal); writing – review and editing (equal). **Alejandra Wu‐Chuang:** Supervision (lead). **Alejandro Cabezas‐Cruz:** Conceptualization (equal); resources (equal); supervision (equal); writing – original draft (supporting); writing – review and editing (lead).

## CONFLICT OF INTEREST STATEMENT

The authors declare no competing interests.

## BENEFIT–SHARING STATEMENT

Research collaboration was developed with scientists from Cuba providing genetic samples. All collaborators are included as co‐authors. The research addresses a priority concern, in this case the incidence of tick‐borne pathogens in Cuba. More broadly, our group is committed to international scientific partnerships, as well as institutional capacity building.

## Supporting information


Figure S1.

Table S1.

Table S2.

Table S3.

Table S4.

Table S5.

Table S6.

Table S7.

Table S8.

Table S9.

Table S10.

Table S11.

Table S12.

Table S13.

Table S14.

Table S15.

## Data Availability

The datasets generated and analyzed during the current study are available on the SRA repository (Bioproject No. PRJNA1028823).
