# Supplementary figures and images for: Dynamic nesting of Anaplasma marginale in the microbial communities of Rhipicephalus microplus
Source: Ecol Evol. 2024 Apr 1;14(4):e11228. doi: 10.1002/ece3.11228 (PMC10985379; doi:10.1002/ece3.11228)

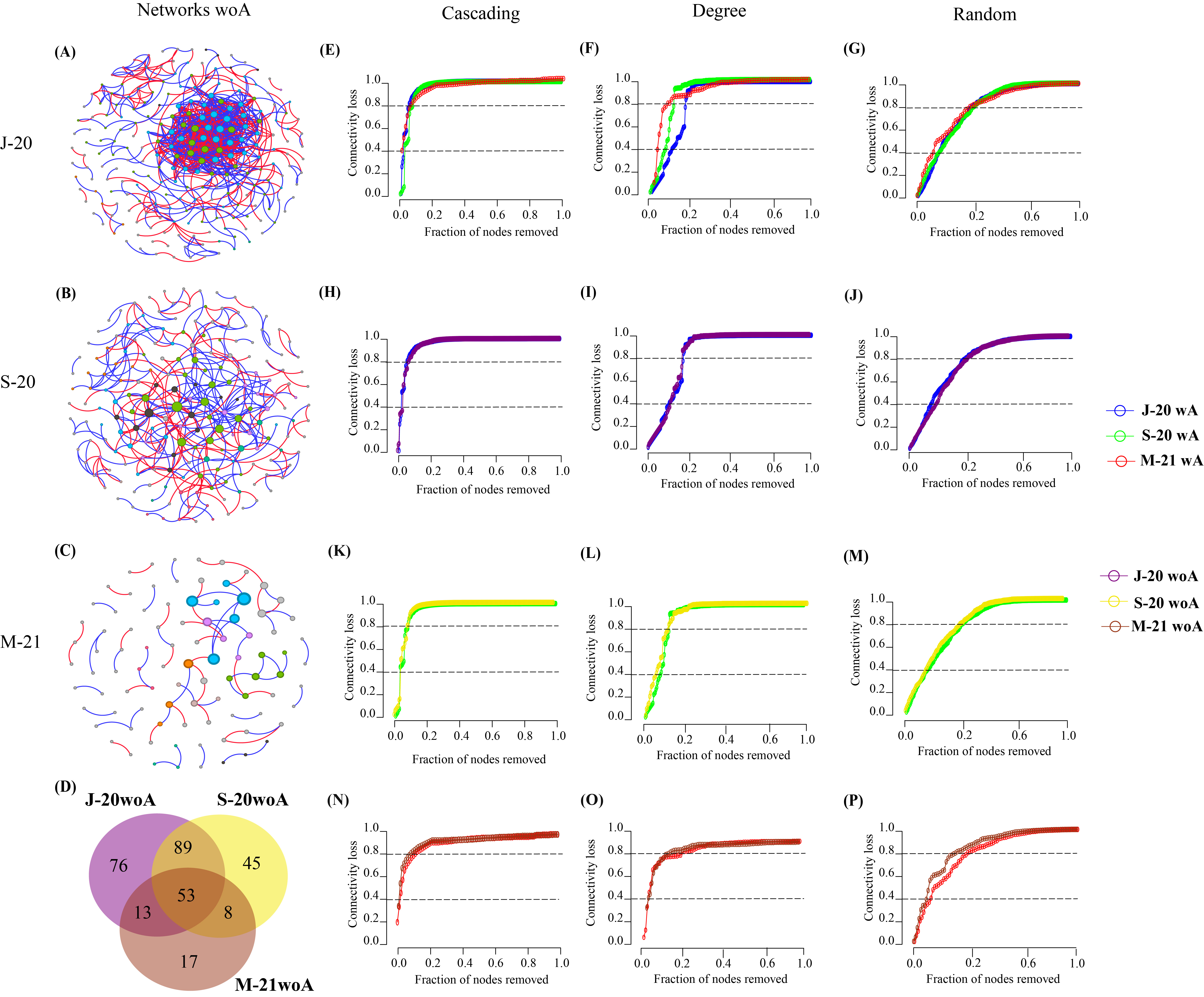

Supplement: Supplementary file 1 — Figure S1. [file ECE3-14-e11228-s011.jpg]
