# Supplementary material for: Dynamic nesting of Anaplasma marginale in the microbial communities of Rhipicephalus microplus
Source: Ecol Evol. 2024 Apr 1;14(4):e11228. doi: 10.1002/ece3.11228 (PMC10985379; doi:10.1002/ece3.11228)
Supplement: Supplementary file 11 — Table S10. [file ECE3-14-e11228-s016.docx]

**Supplementary table S10.** Jaccard index for J-20, S-20 and M-21 networks with *Anaplasma* (wA) for each time.

| **Local centrality measures** | **J-20(wA) *vs*. S-20(wA)** | | | **S-20(wA) *vs*. M-21(wA)** | | | **J-20(wA) *vs*. M-21(wA)** | | |
| --- | --- | --- | --- | --- | --- | --- | --- | --- | --- |
|  | Jacc^a^ | P(≤ Jacc) | P (≥ Jacc) | Jacc^a^ | P (≤ acc) | P (≥ Jacc) | Jacc^a^ | P(≤ Jacc) | P (≥ Jacc) |
| Degree | 0.22 | 0.001 ** | 1 | 0.257 | 0.029 * | 1 | 0.17 | 9e-06 *** | 1 |
| Betweenness centrality | 0.34 | 0.65 | 0.42 | 0.128 | 0.000001*** | 1 | 0.05 | 0 *** | 1 |
| Closeness centrality | 0.23 | 0.002 ** | 1 | 0.250 | 0.018 * | 1 | 0.15 | 0 *** | 1 |
| Eigenvector centrality | 0.21 | 0.0004*** | 1 | 0.250 | 0.018 * | 1 | 0.15 | 0 *** | 1 |
| Hub taxa | 0.22 | 0.0004*** | 1 | 0.250 | 0.018 * | 1 | 0.15 | 0 *** | 1 |
