# Supplementary material for: Dynamic nesting of Anaplasma marginale in the microbial communities of Rhipicephalus microplus
Source: Ecol Evol. 2024 Apr 1;14(4):e11228. doi: 10.1002/ece3.11228 (PMC10985379; doi:10.1002/ece3.11228)
Supplement: Supplementary file 12 — Table S11. [file ECE3-14-e11228-s014.docx]

**Supplementary Table S11.** Jaccard index for J-20, S-20 and M-21 networks without *Anaplasma* (woA) for each time point.

| **Local centrality measures** | **J-20 (woA) *vs*. S-20 (woA)** | | | **S-20 (woA) *vs*. M-21 (woA)** | | | **J-20 (woA) *vs*. M-21 (woA)** | | |
| --- | --- | --- | --- | --- | --- | --- | --- | --- | --- |
|  | Jacc^a^ | P(≤ Jacc) | P (≥ Jacc) | Jacc^a^ | P (≤ acc) | P (≥ Jacc) | Jacc^a^ | P(≤ Jacc) | P (≥ Jacc) |
| Degree | 0.21 | 0.001** | 0.99 | 0.27 | 0.079 ** | 0.94 | 0.16 | 1.1e-05 *** | 0.99 |
| Betweenness centrality | 0.30 | 0.23 | 0.81 | 0.12 | 0 *** | 1 | 0.05 | 0 *** | 1 |
| Closeness centrality | 0.25 | 0.013 * | 0.99 | 0.26 | 0.048* | 0.97 | 0.17 | 5.0e-06 *** | 0.99 |
| Eigenvector centrality | 0.25 | 0.013 * | 0.99 | 0.26 | 0.028* | 0.98 | 0.17 | 5.0e-06 *** | 0.99 |
| Hub taxa | 0.25 | 0.013* | 0.99 | 0.26 | 0.028 * | 0.98 | 0.17 | 5.0e-06 *** | 0.99 |
