# Supplementary material for: Dynamic nesting of Anaplasma marginale in the microbial communities of Rhipicephalus microplus
Source: Ecol Evol. 2024 Apr 1;14(4):e11228. doi: 10.1002/ece3.11228 (PMC10985379; doi:10.1002/ece3.11228)
Supplement: Supplementary file 13 — Table S12. [file ECE3-14-e11228-s010.docx]

**Supplementary Table S12.** Jaccard index for J-20, S-20 and M-21 networks with (wA) *vs.* without (woA) *Anaplasma* in the same time point.

| **Local centrality measures** | **J-20 (wA *vs*. woA)** | | | **S-20 (wA *vs*. woA)** | | | **M-21 (wA *vs*. woA)** | | |
| --- | --- | --- | --- | --- | --- | --- | --- | --- | --- |
|  | Jacc | P(≤ Jacc) | P (≥ Jacc) | Jacc | P (≤ acc) | P (≥ Jacc) | Jacc | P(≤ Jacc) | P (≥ Jacc) |
| Degree | 0.82 | 1 | 0 *** | 0.73 | 1 | 0 *** | 0.72 | 1 | 0 *** |
| Betweenness centrality | 0.77 | 1 | 0 *** | 0.64 | 1 | 0 *** | 0.54 | 0.98 | 0.03 * |
| Closeness centrality | 0.73 | 1 | 0 *** | 0.73 | 1 | 0 *** | 0.72 | 1 | 0 *** |
| Eigenvector centrality | 0.73 | 1 | 0 *** | 0.65 | 1 | 0 *** | 0.72 | 1 | 0 *** |
| Hub taxa | 0.73 | 1 | 0 *** | 0.65 | 1 | 0 *** | 0.72 | 1 | 0 *** |
