# Supplementary material for: Dynamic nesting of Anaplasma marginale in the microbial communities of Rhipicephalus microplus
Source: Ecol Evol. 2024 Apr 1;14(4):e11228. doi: 10.1002/ece3.11228 (PMC10985379; doi:10.1002/ece3.11228)
Supplement: Supplementary file 15 — Table S14. [file ECE3-14-e11228-s007.docx]

**Supplementary Table S14.** Fraction of nodes removed to achieve a 40% and 80% loss of connectivity between nodes in the networks in the presence (wA) and after removal (woA) of *Anaplasma*.

|  | **Fraction of nodes removed to achieve a 40% loss of connectivity** | | | | | | **Fraction of nodes removed to achieve a 80% loss of connectivity** | | | | | |
| --- | --- | --- | --- | --- | --- | --- | --- | --- | --- | --- | --- | --- |
| **Parameters** | **J-20**  **(wA)** | **S-20**  **(wA)** | **M-21**  **(wA)** | **J-20**  **(woA)** | **S-20**  **(woA)** | **M-21**  **(woA)** | **J-20**  **(wA)** | **S-20**  **(wA)** | **M-21**  **(wA)** | **J-20**  **(woA)** | **S-20**  **(woA)** | **M-21**  **(woA)** |
| **Betweenness** | 0.13 | 0.15 | 0.09 | 0.13 | 0.14 | 0.08 | 0.36 | 0.36 | 0.34 | 0.33 | 0.35 | 0.32 |
| **Cascading** | 0.13 | 0.13 | 0.03 | 0.13 | 0.13 | 0.02 | 0.22 | 0.20 | 0.11 | 0.21 | 0.20 | 0.10 |
| **Degree** | 0.08 | 0.05 | 0.01 | 0.08 | 0.05 | 0.007 | 0.15 | 0.14 | 0.12 | 0.15 | 0.14 | 0.13 |
| **Random** | 0.0 3 | 0.03 | 0.012 | 0.016 | 0.03 | 0.011 | 0.08 | 0.08 | 0.07 | 0.08 | 0.07 | 0.06 |
