# Supplementary material for: Dynamic nesting of Anaplasma marginale in the microbial communities of Rhipicephalus microplus
Source: Ecol Evol. 2024 Apr 1;14(4):e11228. doi: 10.1002/ece3.11228 (PMC10985379; doi:10.1002/ece3.11228)
Supplement: Supplementary file 16 — Table S15. [file ECE3-14-e11228-s008.docx]

**Supplementary Table S15.** Largest connected component (LCC) and average path length (APL) values in the presence (wA) and after removal (woA) of *Anaplasma* from the networks.

| **Nodes** | **LCC (wA)** | | | **APL (wA)** | | | **LCC (woA)** | | | **APL (woA)** | | |
| --- | --- | --- | --- | --- | --- | --- | --- | --- | --- | --- | --- | --- |
|  | **J-20 (wA)** | **S-20**  **(wA)** | **M-21**  **(wA)** | **J-20 (wA)** | **S-20**  **(wA)** | **M-21**  **(wA)** | **J-20 (woA)** | **S-20**  **(woA)** | **M-21**  **(woA)** | **J-20 (woA)** | **S-20**  **(woA)** | **M-21**  **(woA)** |
| **5** | 217 | 195 | 27 | 4.52 | 5.72 | 3.96 | 202 | 184.9 | 33 | 4.79 | 5.16 | 5.58 |
| **10** | 224 | 205 | 31.5 | 4.54 | 5.53 | 4.86 | 203 | 185.1 | 36 | 4.69 | 5.14 | 5.99 |
| **25** | 226 | 206 | 56 | 5.01 | 5.36 | 6.23 | 212.7 | 191 | 39 | 4.76 | 5.12 | 5.94 |
| **50** | 229 | 206 | 75 | 4.63 | 5.13 | 6.19 | 217.8 | 193 | 79 | 4.63 | 4.77 | 6.27 |
| **75** | 239 | 212 | 89 | 4.73 | 4.93 | 5.90 | 227.9 | 199.4 | 80 | 4.64 | 4.85 | 6.08 |
| **100** | 240 | 209 | 87.2 | 4.34 | 4.61 | 4.72 | 224.1 | 193.9 | 89 | 4.29 | 4.48 | 4.89 |

* Nodes were incrementally added in sections ranging from 5 to 100.
